# The effect of GMFCS level, age, sex, and dystonia on multi-dimensional outcomes after selective dorsal rhizotomy: prospective observational study

**DOI:** 10.1007/s00381-021-05076-0

**Published:** 2021-02-18

**Authors:** Conor Scott Gillespie, Alan Matthew George, Benjamin Hall, Steven Toh, Abdurrahman Ismail Islim, Dawn Hennigan, Alder Hey Physiotherapy Group, Ram Kumar, Benedetta Pettorini

**Affiliations:** 1 Present Address: Department of Neurosurgery, Alder Hey Children’s Hospital NHS Trust, Liverpool, UK; 2 Institute of Systems, Molecular and Integrative Biology, University of Liverpool, Biosciences Building, Crown Street, Liverpool, L69 7BE UK; 3 Institute of Inflammation and Ageing, College of Medical and Dental Sciences, University of Birmingham, Birmingham, UK; 4 Aintree University Hospitals NHS Foundation Trust, Liverpool, UK; 5 School of Medicine, University of Liverpool, Liverpool, UK; 6 Royal Liverpool and Broadgreen Hospitals NHS Trust, Liverpool, UK; 7 Department of Physiotherapy, Alder Hey Children’s Hospital NHS Trust, Liverpool, UK

**Keywords:** SDR, Cerebral palsy, GMFCS, GMFM

## Abstract

**Purpose:**

Investigate the effect of age category (1–9 years vs 10–18 years), sex, Gross Motor Function Classification System (GMFCS) level, and presence of dystonia on changes in eight function test parameters 24 months after selective dorsal rhizotomy (SDR).

**Methods:**

Prospective, single-center study of all children aged 3–18 years with bilateral cerebral palsy with spasticity who underwent SDR at a tertiary pediatric neurosurgery center between 2012 and 2019. A linear mixed effects model was used to assess longitudinal changes.

**Results:**

From 2012 to 2019, 42 children had follow-up available at 24 months. Mean GMFM-66 scores increased after SDR (mean difference 5.1 units: 95% CI 3.05–7.13, *p* < 0.001). Statistically significant improvements were observed in CPQoL, PEDI Self-care and Mobility, 6MWT, Gillette, and MAS scores. There was no significant difference in the improvements seen for age category, sex, GMFCS level, and presence of dystonia for most of the parameters tested (5/8, 6/8, 5/8, and 6/8 respectively).

**Conclusion:**

SDR may improve gross and fine motor function, mobility and self-care, quality of life, and overall outcome based on extensive scoring parameter testing at 24 months. Atypical patient populations may benefit from SDR if appropriately selected. Multi-center, prospective registries investigating the effect of SDR are required.

## Introduction

Cerebral palsy (CP) and its subsequent impact can delay the achievement of clinical, functional, and developmental milestones [1]. The prevalence of cerebral palsy is approximately 2 in 1000 live births, of which 80% of children have spastic cerebral palsy [2].

Movement disorders associated with cerebral palsy adversely affect mobility and quality of life, and can be categorized into hypertonia (spasticity, dystonia, athetosis, and chorea) and hypotonia [3]. Untreated spasticity can cause pain and discomfort, limit mobility, and result in skeletal deformities such as joint luxation or subluxation [3].

Spasticity can be managed with surgical intervention. In selective dorsal rhizotomy (SDR), partial transection of dorsal rootlets reduces the sensory input into reflex arcs responsible for increased muscle tone, while preserving voluntary movement [4]. This can be used singularly or in combination with other treatments to improve quality of life, by improving functional movement capabilities and reducing pain [5]. Other treatments available include botulinum toxin injections, which may lead to a transient and incomplete response, and intrathecal baclofen (ITB) therapy [6].

Currently, the largest evidence base outlining eligibility for SDR is the National institute for Health and Care Excellence (NICE) criteria, which recommend consideration of SDR for CP in children aged 3–9 years old with GMFCS level II or III [5]. It is indicated for children who would benefit from a significant improvement in motor function and quality of life after undergoing SDR together with physiotherapy. SDR has yielded consistent improvements over time in function assessed using Gross Motor Function Measure (GMFM-66) and quality of life assessed using the Cerebral Palsy Quality of Life Questionnaire (CP-QoL) over a 2-year follow-up [7, 8].

The selection criteria and efficacy of SDR are still an issue of ongoing debate [9], due to the lack of randomized clinical trials (RCTs) and control groups in most studies [8, 10]. There is a growing body of evidence that suggest promising outcomes for SDR in children with GMFCS levels IV and V [11], and three randomized control trials (RCTs) identified in a meta-analysis of SDR all included GMFCS level IV patients, but this is still to be established [12].

In addition, there is a lack of evidence in patients over the age of 9 years and those with mixed spasticity and dystonia (with a surgical aim to improve spasticity), as these patients are often excluded from studies investigating SDR [8]. Most children with hypertonia have both spasticity and dystonia co-existing to a certain extent [13].

The long-term effects of SDR are also unclear, and a recent Cochrane review of long-term outcomes after SDR (follow-up of 10 years or more) failed to identify a documented functional improvement compared to routine therapy [14]. However, some studies have highlighted its potential short- and long-term functional benefits [15, 16]. Many studies have reported equivocal results [17].

Analysis of the effect of SDR on gross and fine motor function, overall mobility, and quality of life has mainly been employed through the use of GMFM-66 and CPQoL scores [8, 10, 18]; and thus, measures of its effect utilizing other validated scoring tests are unclear. Other validated scores exist that assess gross and fine motor function, self-care, quality of life, and overall well-being in CP [19, 20]. The effect of SDR on these scoring systems has yet to be evaluated comprehensively. Improvements/changes seen in these validated scoring systems could provide multi-dimensional outcomes and a greater insight into the overall effect of SDR on quality of life [19, 20].

## Objectives

The primary objectives of the study were to investigate the effect of age category (1–9 years vs 10–18 years), sex, GMFCS level, and dystonia on changes in scoring parameters, to establish which groups received the greatest benefits (if any) after undergoing SDR. The secondary objectives of the study were to extensively evaluate the effect of SDR at 24 months after surgery on gross and fine motor function, quality of life, self-care, and overall well-being through assessment of eight different assessment tests.

## Methods

### Study design

We carried out a prospective observational single-center study in accordance with the STROBE statement [21], of all cases of SDR operated between 2012 and 2019, at the Department of Neurosurgery, Alder Hey Children’s Hospital, a regional tertiary pediatric neurosurgical center for SDR in England, UK. The center was experienced in delivering SDR in the context of other treatment options for spasticity and dystonia including botulinum toxin injections, ITB, selective peripheral neurectomy, and deep brain stimulation. Audit approval was obtained from the Neurosurgical Department clinical audit team prior to commencement of the study.

### Participants

Eligible children had bilateral spastic cerebral palsy that limited functional capabilities and were suitable candidates for surgery. Patients of GMFCS levels I, II, III, IV, and V, age up to 18 years, patients with mixed spasticity and dystonia (graded by the Barry-Albright Dystonia Scale (BAD)), and operated on within the time period were all eligible for the study. This was because these patients were considered good surgical candidates after multi-disciplinary review and could potentially benefit from the procedure. Patients were excluded if they had progressive neurological conditions or were operated on outside of the study period.

### Baseline characteristics

The baseline clinical characteristics recorded for each patient included age at SDR, sex, age category (3–9 years or 10–18 years), and presence or absence of dystonia defined using the hypertonia assessment tool (HAT) [22], with a score of at least one being classified as dystonia being present. The domains assessed before SDR and at each follow-up assessment were GMFM-66 score, Cerebral Palsy Quality of Life (CPQoL) questionnaire-primary caregiver (parent), Pediatric Evaluation of Disability Inventory (PEDI) (self-care and mobility components), Timed up and go test (TUG), 6-Min Walk Test (6MWT), Gillette Functional Assessment Questionnaire (FAQ), and Modified Ashworth Scale (MAS). Summaries of the components of each test assessed are outlined in Table 1.

**Table 1:** Summary of tests performed at baseline and each follow-up assessment after SDR. *CP* cerebral palsy, *ROM* range of motion

Test name	Test components	Scoring parameters
Gross Motor Function Measure (GMFM)-66	66 Item subset of GMFM-88 used to describe gross motor function of children with CP of varying abilities. Five domains: Lying and Rolling, Sitting, Crawling and Kneeling, Standing, and Walking, Running, and Jumping	For each task: 0 = does not initiate 1 = initiates 2 = partially completes 3 = completes Higher score= better function
Cerebral palsy Quality of Life (QoL) questionnaire-Primary Caregiver (parent)	Questionnaire given to primary caregiver to assess quality of life for patients with cerebral palsy. Domains assessed: Social wellbeing and acceptance Feelings about functioning Participation and physical health Emotional well-being and self-esteem Access to services Pain and impact of disability Family health	Scale of 1–9 on how the caregiver thinks the child feels (1 = very unhappy, 9 = very happy) Higher score = higher perceived quality of life
Pediatric Evaluation of Disability Inventory (PEDI) Self-care and mobility components	Interview-based assessment used to monitor self-care, mobility, and social abilities of children with CP (up to age 7.5 years) Domains: Can the child perform/carry out: Daily activities, mobility, social and cognitive function, and responsibility (independence)	Scaled scores dependent on capability Higher score = higher degree of self-care/mobility maintained
Timed up and go test (TUG)	Time taken to rise from a chair, walk 3 m, turn around, walk back to chair, and sit down	< 10 s: normal 10–20 s: good mobility, can go out alone, mobile without gait aids > 20 s: problems, cannot go outside alone, requires gait aid Lower score = better function
6-Min Walk test (6MWT)	Maximum distance (m) walked in 6 min, either within a 30-m distance or on a treadmill device	Length in meters times for CP GMFCS level defined by Fitzgerald et al. Higher score = better function
Gillette Functional Assessment Questionnaire (FAQ)	Self-reported measure of locomotor function, with ten-item walking scale and 22 item locomotor activity score using Likert scales	Weighted scoring based on difficulty of task (logits) Higher score = better function
Modified Ashworth scale (MAS)	Measure of spasticity, measures resistance during soft-tissue stretching	0: No increase in muscle tone 1: Slight increase in muscle tone 1+: Slight increase, catch, then minimal resistance 2: Marked increase in tone through ROM 3: Passive movement difficult Affected part rigid in flexion or extension Higher score = greater spasticity

### Surgical technique and follow-up

All cases were performed by a single surgeon (BP), carried out by neurophysiology-guided partial resection of the dorsal (sensory) roots as previously described [23]. Patients were enrolled to 3 months of inpatient physiotherapy followed by post-operative physiotherapy lasting 24 months after surgery. Patients with dystonia continued existing pharmacological treatment if applicable throughout the study period. Follow-up assessments and data collection were conducted by trained physiotherapists and neurosurgeons when appropriate at baseline/pre-operatively, 3 months, 6 months, 12 months, 24 months, and 5 years post-SDR if follow-up was available.

### Study outcomes

The primary outcome measure was the impact of GMFCS level, sex, age category, and presence of dystonia on the changes to scoring tests after SDR. The secondary outcome measures were the changes in GMFM-66, CPQoL, PEDI Self-care and mobility score, TUG and 6MWT test, Gillette FAQ scores, and MAS scale after SDR.

### Statistical analysis

To evaluate longitudinal changes in the scoring parameters over time until 24 months after SDR and to account for attrition rates in the study or differential follow-up, we carried out a linear mixed effects model, in which the patient was the random effect, with time after SDR, sex, age category (1–9 vs 10 and over), and presence of dystonia the fixed effect. A restricted log likelihood was determined to analyze the model with the best fit for each variable (Compound symmetry, AR: heterogenous or Unstructured). Differences over time were assessed by fitting an interaction term in the model with a likelihood ratio test. As previous studies have used 24 months after SDR as an appropriate time point, results were scaled to this with 95% confidence intervals (CIs). Model fit and assumptions were examined through the use of residual plots. We assumed a *p* value of < 0.05 for statistical significance. To assess the effects of each interaction variable, we incorporated GMFCS level, age category, sex, and presence of dystonia into the mixed model for each scoring parameter tested. Spaghetti and smoothed conditional means plots were utilized to represent the longitudinal trends in scores over time after SDR. Data was analyzed using R v4.02 and SPSS v25.0.

## Results

### Patients and demographics

Between 2012 and 2019, 145 children between the ages of 3 and 18 underwent SDR. Of these, six were excluded due to having progressive neurological conditions, leaving 139 children eligible for the study and included in the analysis. Patient demographics are summarized in Table 2. The median age at SDR was 7 years of age (mean 6 years and 2 months, standard deviation (SD) 3 years 4 months). Most patients treated with SDR were female (*n* = 83, 59.7%). The male: female ratio was approximately 1:1.5. In total, 17.5% of patients had mixed spasticity and dystonia (*N* = 22/126). Eight children underwent orthopedic surgery before SDR (7.4%, *n* = 8/108). The median BAD scale at baseline for each patient was 0 (mean 3.86, IQR 0–5).

**Table 2:** Baseline characteristics

Age at SDR (years)	
Median (SD)	7.0 (3.29)
Range	15 (3–18)
Gender	*N* (%)
Boys	56 (40.3%)
Girls	83 (59.7%)
GMFCS level	*N* (%)
I	3 (2.1%)
II	23 (15.8%)
III	65 (44.5%)
IV	46 (31.5%)
V	9 (6.2%)
Presenting with dystonia	N (%)
Yes	22 (17.5%)
No	104 (82.5%)
Number of patients with follow-up data	*N* (%)
Baseline	139 (100%)
3 months	72 (51.7%)
6 months	64 (46.0%)
12 months	60 (43.1%)
24 months	42 (30.2%)
5 years	6 (4.3%)
Age category	*N* (%)
3–9 years	116 (83.5%)
10–18 years	23 (16.5%)
Orthopedic surgery	*N* (%)
No	83 (76.9%)
Before SDR	8 (7.4%)
After SDR	17 (15.7%)

The most frequent GMFCS level before surgery was III (45%, *n* = 61/135). All patients had nerve rootlets cut in L2-S1, and the surgical procedure was similar to previously published studies.

### Follow-up

Sixty patients had follow-up lasting 12 months (*n* = 60/139, 40%), and 42 patients had follow-up lasting 24 months (*n* = 42/139, 30%). Of the 22 patients with mixed spasticity and dystonia, 8 had follow-up lasting 24 months (*n* = 8/22, 36%). In total, 15.7% underwent further orthopedic surgery after SDR (*n* = 17/108).

### Scoring parameter changes

**Fig. 1:**
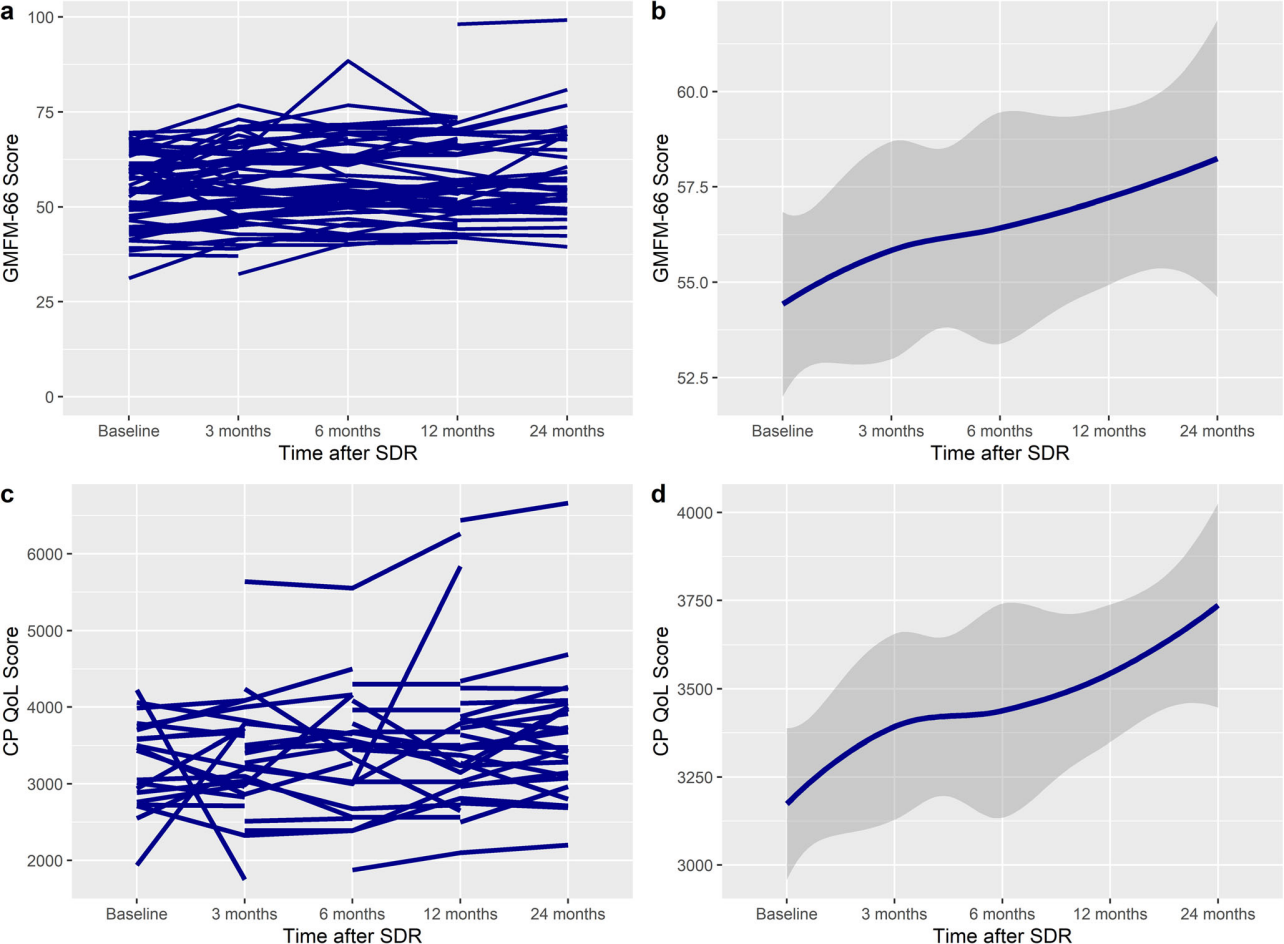

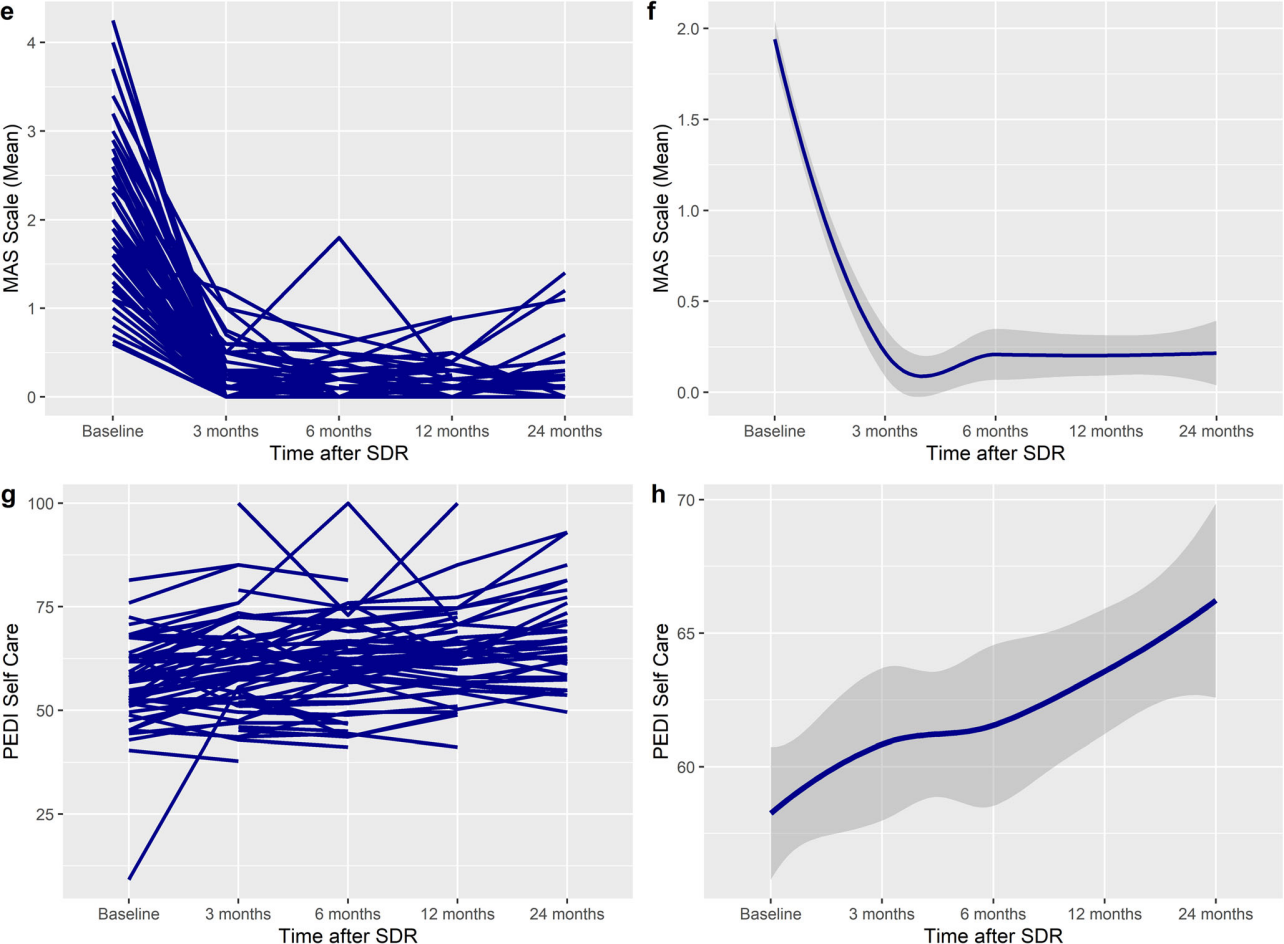
Spaghetti plot of **a** GMFM-66, **c** CPQoL (primary caregiver), **e** Modified Ashworth Scale (MAS), and **g** PEDI Self-care score over time, demonstrating an improvement after SDR. **b**, **d**, **f**, and **h** show the smoothed conditional means with standard error over time (y ≠ 0)

GMFM-66 scores increased linearly after SDR to 24 months after surgery (Fig. 1). The mean difference at 24 months was significant, with an overall increase of 5.1 units (95% CI 3.05–7.13, *p* < 0.001) (Table 3). There was no difference in the increase between different GMFCS levels (Table 4) (*p*_interaction_ = 0.100), sex (*p*_interaction_=0.183), age category (*p*_interaction_=0.153), or patients with dystonia (*p*_interaction_=0.07) (Table 5).

**Table 3:** Estimated marginal (*EM*) mean GMFM-66, CPQoL, PEDI Mobility, PEDI Self-care, TUG, 6MWT, Gillette, and MAS scores over time after SDR. *SD* standard deviation, *CI* confidence interval

Time after selective dorsal rhizotomy (SDR)	Number of patients	GMFM-66 Mean (SD)	Mean difference from baseline (95% CI)	*p* value
Baseline/pre-op	99	54.2 (11.2)	N/A	-
3 months	68	56.0 (12.3)	1.74 (0.1, 3.4)	0.040
6 months	62	57.2 (12.5)	3.10 (1.3, 4.8)	0.001
1 year	59	57.4 (12.3)	3.12 (1.6, 4.7)	< 0.001
2 years	41	59.3 (13.6)	5.09 (3.1, 7.1)	< 0.001
5 years	5	60.0 (31.8)	4.92 (2.3, 7.5)	< 0.001
Time after selective dorsal rhizotomy (SDR)	Number of patients	CPQoL Mean (SD)	Mean difference from baseline (95% CI)	*p* value
Baseline/pre-op	56	3175.0 (660.8)	N/A	-
3 months	37	3392.4 (667.7)	217 (− 57, 492)	0.117
6 months	28	3447.1 (620.2)	272 (− 11, 555)	0.059
1 year	45	3578.7 (846.6)	404 (117, 690)	0.007
2 years	29	3745.4 (821.8)	570 (238, 903)	0.001
5 years	4	3365.4 (543.6)	190 (− 2308, 2689)	0.608
Time after selective dorsal rhizotomy (SDR)	Number of patients	PEDI Self-care Mean (SD)	Mean difference from baseline (95% CI)	*p* value
Baseline/pre-op	90	57.81 (13.3)	N/A	-
3 months	68	61.71 (10.7)	3.90 (1.81, 6.00)	< 0.001
6 months	61	62.60 (10.2)	4.79 (2.19, 7.39)	< 0.001
1 year	54	63.15 (9.6)	5.34 (2.44, 8.25)	< 0.001
2 years	39	66.43 (10.6)	8.62 (4.98, 12.27)	< 0.001
5 years	5	71.40 (6.3)	13.59 (7.72, 19.47)	<0.001
Time point after selective dorsal rhizotomy (SDR)	Number of patients	PEDI Mobility score Mean (SD)	Mean difference from baseline (95% CI)	*p* value
Baseline/pre-op	94	49.01 (13.7)	N/A	-
3 months	72	51.91 (12.7)	− 1.28 (− 3.34, 0.78)	0.222
6 months	64	53.82 (13.4)	0.62 (− 1.60, 2.85)	0.581
1 year	56	54.88 (12.0)	1.69 (− 0.82, 4.19)	0.184
2 years	38	57.60 (12.1)	4.41 (1.67, 7.14)	0.002
5 years	5	56.75 (5.4)	3.55 (− 0.33, 7.43)	0.072
Time point selective dorsal rhizotomy (SDR)	Number of patients	TUG (s) Mean (SD)	Mean difference from baseline (95% CI)	*p* value
Baseline/pre-op	71	31.61 (34.5)	N/A	-
3 months	47	31.29 (27.4)	− 0.32 (− 7.26, 6.62)	0.927
6 months	44	26.06 (22.6)	− 5.56 (− 13.56, 2.50)	0.172
1 year	45	26.31 (30.2)	− 5.30 (− 15.35, − 4.75)	0.299
2 years	33	21.57 (36.7)	− 10.05 (− 23.68, 3.58)	0.147
5 years	4	23.90 (15.0)	− 7.71 (− 26.51, 11.08)	0.378
Time point selective dorsal rhizotomy (SDR)	Number of patients	6-minwalk test (6MWT) Mean (SD)	Mean difference from baseline (95% CI)	*p* value
Baseline/pre-op	82	206.6 (118.6)	N/A	-
3 months	50	173.0 (101.8)	− 33.6 (− 55.2, − 12.0)	0.003
6 months	47	208.4 (82.3)	1.8 (− 22.9, 26.5)	0.886
1 year	47	222.2 (96.7)	15.6 (− 14.4, 45.6)	0.305
2 years	34	261.2 (102.0)	54.6 (17.2, 92.0)	0.005
5 years	4	314.9 (59.6)	108.3 (40.4, 176.7)	0.004
Time point selective dorsal rhizotomy (SDR)	Number of patients	Gillette Functional Assessment Questionnaire Mean (SD)	Mean difference from baseline (95% CI)	*p* value
Baseline/pre-op	103	5.12 (2.3)	N/A	-
3 months	72	5.39 (2.2)	0.27 (-0.17, 0.55)	0.065
6 months	63	5.66 (2.0)	0.54 (0.16, 0.92)	0.005
1 year	58	5.81 (2.0)	0.69 (0.24, 1.14)	0.03
2 years	37	5.97 (1.9)	0.85 (0.26, 1.44)	0.005
5 years	5	6.81 (0.9)	1.68 (0.78, 2.58)	0.001
Time point selective dorsal rhizotomy (SDR)	Number of patients	Modified Ashworth Scale (MAS) Mean (SD)	Mean difference from baseline (95% CI)	*p* value
Baseline/pre-op	120	1.95 (0.62)	N/A	-
3 months	74	0.17 (0.59)	− 1.781 (− 1.91, − 1.65)	< 0.001
6 months	66	0.15 (0.58)	− 1.795 (− 1.93, − 1.66)	< 0.001
1 year	57	0.11 (0.57)	− 1.840 (− 1.99, − 1.70)	< 0.001
2 years	38	0.17 (0.54)	− 1.774 (− 1.94, − 1.61)	< 0.001
5 years	3	0.17 (0.47)	− 1.770 (− 2.30, − 1.24)	< 0.001

**Table 4:** Changes in estimated marginal (*EM*) mean GMFM-66, CPQoL, PEDI Self-care, PEDI Mobility, TUG, 6MWT, Gillette, and MAS scores stratified by GMFCS level. *GMFCS level V used as comparator variable. *CI* confidence interval, *SD* standard deviation, *FAQ* Functional Assessment Questionnaire. - not available

GMFM-66	Before SDR (SD)	24 months after SDR (SD)	95% CI
GMFCS level I	78.2 (10.7), *n* = 2	88.0 (8.5), *n* = 1	63.0–93.3, 76.0–100.0
GMFCS level II	67.5 (7.6), n=13	79.2 (12.5), *n* = 5	63.2–71.7, 69.0–89.4
GMFCS level III	57.0 (8.2), *n* = 55	60.0 (8.0), *n* = 23	54.8–59.3, 57.1–62.9
GMFCS level IV	42.5 (7.8), *n* = 21	48.0 (8.3), *n* = 12	39.2–45.9, 44.1–51.9
GMFCS level V*	-	-	-
All patients	51.3 (19.1), *n* = 91	68.8 (16.3), *n* = 41	47.3–55.3, 64.7–72.9
CP-QoL Parent (overall)	Before SDR (SD)	24 months after SDR (SD)	95% CI
GMFCS level I	3405.9 (692.4), *n* = 2	-	-
GMFCS level II	3228.7 (684.7), *n* = 14	-	-
GMFCS level III	3282.8 (666.5), *n* = 34	3730.0 (816.6), *n* = 22	3053.9–3511.8, 3378.2–4081.6
GMFCS level IV	2649.9 (681.3), *n* = 7	4018.0 (844.3), *n* = 7	2134.7–3165.1, 3373.4–4662.6
GMFCS level V*	-	-	-
All patients	3141.8 (1120.4), *n* = 57	3874.0 (978.5), *n* = 29	2844.4–3439.3, 3506.8–4241.1
PEDI Self-care	Before SDR (SD)	24 months after SDR (SD)	95% CI
GMFCS level I	41.4 (11.3), *n* = 2	80.1 (14.1), *n* = 2	25.6–57.2, 57.2–103.0
GMFCS level II	65.9 (10.5), *n* = 15	64.6 (16.3), *n* = 5	60.5–71.3, 50.0–79.2
GMFCS level III	60.9 (10.6), *n* = 50	70.7 (11.5), *n* = 25	57.9–64.0, 66.4–74.9
GMFCS level IV	51.5 (9.6), *n* = 21	58.2 (10.5), *n* = 10	47.3–55.7, 52.8–63.6
GMFCS level V*	*N* = 2	-	-
All patients	48.9 (22.8), *n* = 90	68.4 (33.9), *n* = 42	44.3–53.7, 61.4–75.4
PEDI Mobility	Before SDR (SD)	24 months after SDR (SD)	95% CI
GMFCS level I	59.8 (9.9), *n* = 2	64.1 (15.8), *n* = 2	45.9–73.6, 41.6–86.5
GMFCS level II	68.1 (9.7), *n* = 15	74.2 (9.4), *n* = 5	63.2–72.9, 65.8–82.6
GMFCS level III	57.7 (9.9), *n* = 50	63.3 (10.3), *n* = 24	55.0–60.4, 59.0–67.5
GMFCS level IV	40.6 (9.0), *n* = 25	46.0 (10.0), *n* = 12	36.9–44.2, 40.3–51.7
GMFCS level V*	-	-	-
All patients	48.2 (20.1), *n* = 92	57.7 (23.6), *n* = 43	44.1–52.4, 50.3–65.0
TUG (s)	Before SDR (SD)	24 months after SDR (SD)	95% CI
GMFCS level I	9.4 (22.7), *n* = 2	5.8 (28.7), *n* = 2	− 35.8 to 54.7, − 51.7 to 63.4
GMFCS level II	11.9 (23.0), *n* = 17	4.2 (29.6), *n* = 4	− 5.2 to 26.5, − 55.3to 63.7
GMFCS level III	38.5 (4.9), *n* = 44	27.2 (7.7), *n* = 24	28.7–48.2, 11.8–42.6
GMFCS level IV	43.2 (11.6), *n* = 8	27.1 (15.5), *n* = 6	20.2–66.3, − 4.0 to 58.1
GMFCS level V*	-	-	-
All patients	25.4 (6.8), *n* = 71	16.1 (8.7), *n* = 36	11.9–39.0, − 6.3 to 38.5
6MWT (m)	Before SDR (SD)	24 months after SDR (SD)	95% CI
GMFCS level I	333.1 (94.3), *n* = 2	397.9 (94.5), *n* = 2	200.5–465.7, 263.2–532.6
GMFCS level II	314.4 (23.1), *n* = 17	402.9 (196.3), *n* = 7	268.4–360.4, 253.5–552.3
GMFCS level III	191.9 (95.2), *n* = 52	257.4 (88.2), *n* = 24	165.5–218.2, 186.8–241.5
GMFCS level IV	89.4 (94.2), *n* = 11	121.3 (88.4), *n* = 7	33.0–146.0, 54.1–188.4
GMFCS level V*	-	-	-
All patients	232.2 (174.8), *n* = 82	294.9 (168.9), *n* = 40	193.8–270.6, 241.1–348.6
Gillette FAQ	Before SDR (SD)	24 months after SDR (SD)	95% CI
GMFCS level I	8.5 (1.7), *n* = 2	9.2 (2.4), *n* = 2	6.2–10.8, 5.7–12.6
GMFCS level II	7.5 (1.5), *n* = 14	9.2 (3.8), *n* = 5	6.7–8.4, 5.4–10.9
GMFCS level III	6.3 (1.5), *n* = 55	7.1 (1.7), *n* = 19	5.8–6.7, 6.5–7.8
GMFCS level IV	2.1 (1.6), *n* = 30	2.9 (1.7), *n* = 16	1.6–2.7, 2.0–3.8
GMFCS level V*	*N* = 2	-	-
All patients	5.1 (4.1), *n* = 103	6.9 (4.6), *n* = 42	4.4–5.8, 5.7–7.6
MAS	Before SDR (SD)	24 months after SDR (SD)	95% CI
GMFCS level I	1.61 (0.4), *n* = 3	0.14 (0.7), *n* = 2	0.96 2.26, − 0.90 to 1.18
GMFCS level II	1.56 (0.4), *n* = 19	0.15 (1.0), *n* = 4	1.30–1.81, − 1.08 to 0.78
GMFCS level III	1.85 (0.8), *n* = 60	0.10 (0.5), *n* = 24	1.70–1.99, − 1.1 to 0.31
GMFCS level IV	2.42 (0.6), *n* = 34	0.47 (0.4), *n* = 13	2.2–2.6, 0.19–0.75
GMFCS level V*	*N* = 4	-	-
All patients	1.78 (1.0), *n* = 116	0.14 (1.3), *n* = 43	1.56–1.97, − 0.19 to 0.47

**Table 5:** Interactions between improvements in scores at 24 months and categorical variables (GMFCS level, age categories 1–9 and 10–18 years, sex, and presence of dystonia. *Significant *p* value (< 0.05) indicates a differential increase/improvement between categories. A non-significant *p* value (*p* > 0.05) indicates a non-significant difference between increase/improvement in scores for each category

Interactions between change in score and categorical variables* (*p*_interaction_) (improvement in units at 24 months)								
	GMFM-66	CPQoL	PEDI Self-care	PEDI Mobility	TUG (s)	6MWT (m)	Gillette FAQ	MAS
GMFCS level	0.100	0.005	< 0.001	0.021	0.227	0.308	0.167	0.748
Age categories (10-18 vs 1-9)	0.153	0.001	0.008	0.168	0.033	0.116	0.223	0.848
Gender	0.183	0.387	0.008	0.317	0.043	0.551	0.934	0.302
Dystonia	0.070	0.256	0.061	0.016	0.007	0.211	0.480	0.335

### CPQoL scores

**Fig. 2:**
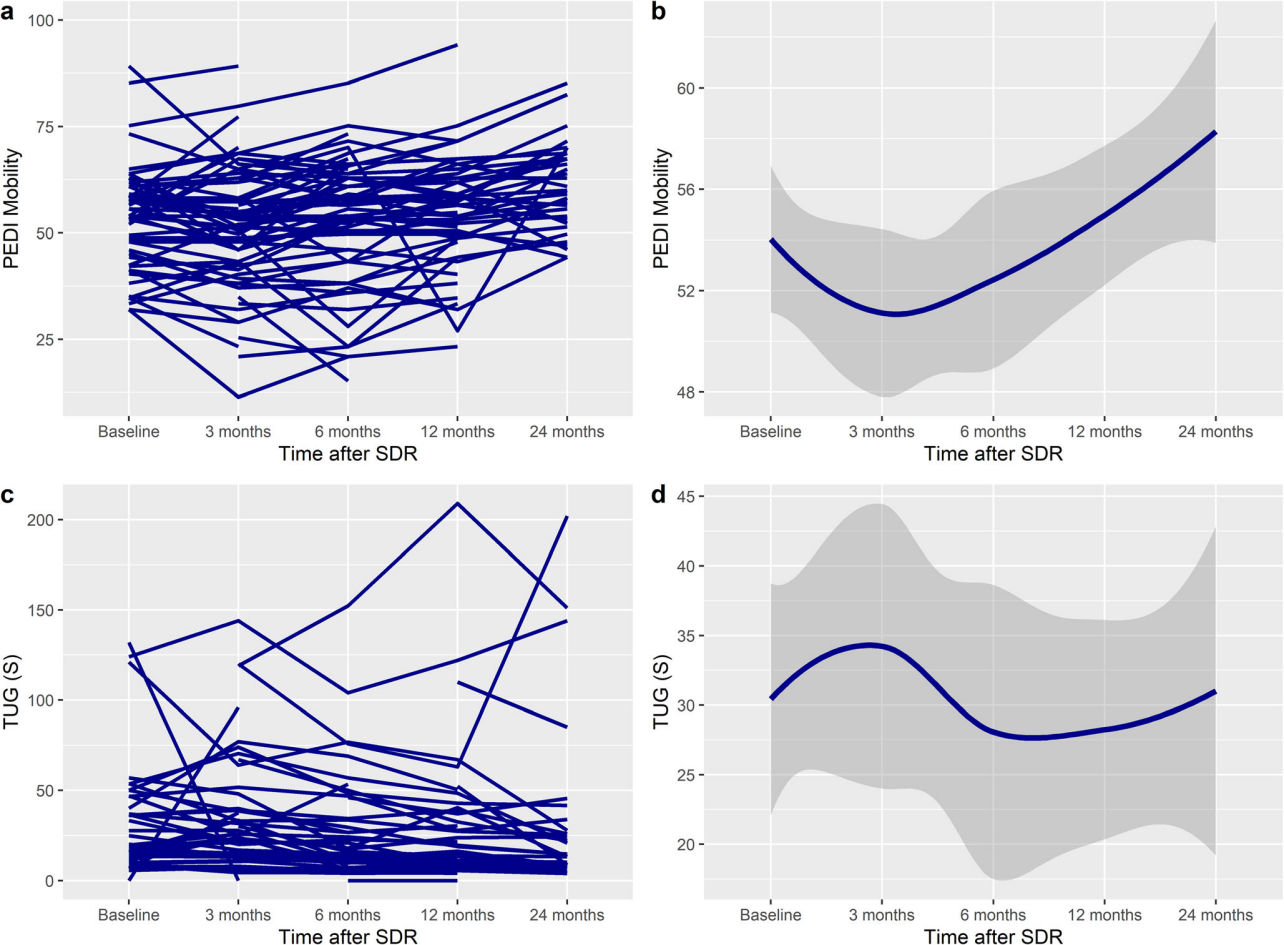

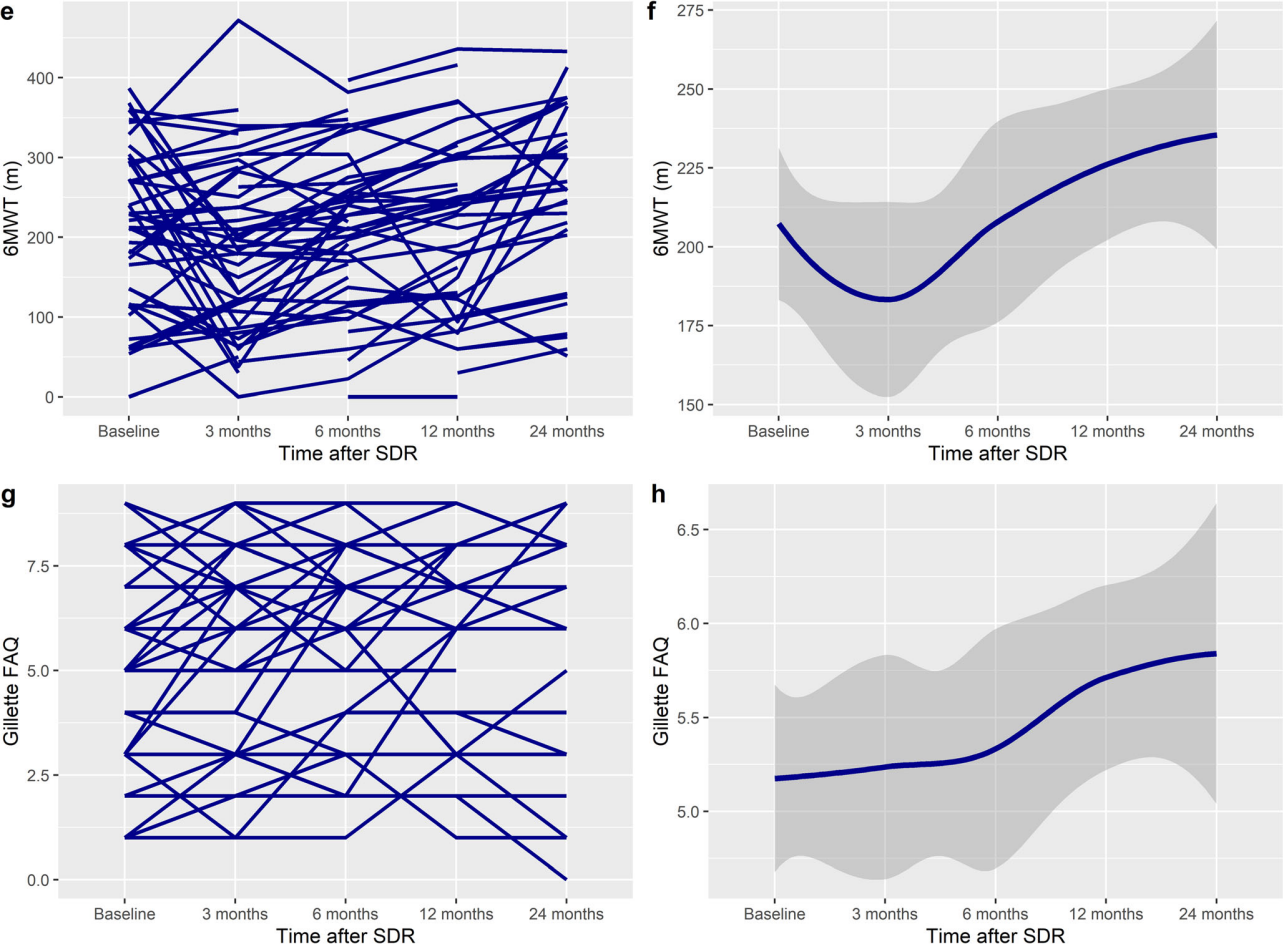
Spaghetti plot of **a** PEDI Mobility, **c** TUG, **e** 6MWT, and **g** Gillette FAQ scores over time, demonstrating an improvement after SDR. In 6MWT and Gillette, there is an initial decrease in scores, followed by an increase. **b**, **d**, **f**, and **h** show the smoothed conditional means with standard error over time (y ≠ 0)

CPQoL scores increased linearly after SDR (Fig. 1), with a significant improvement at 24 months, with an overall increase of 570 units (95% CI 283–930, *p* < 0.001) (Table 3). GMFCS levels III and IV patients scores increased more over time than those with GMFCS levels I or II (*p*_interaction_ = 0.005). There was no difference in the increase for sex (*p*_interaction_=0.387) or patients with dystonia (*p*_interaction_=0.256). The increase in CPQoL was significantly higher in patients in age group 10–18 than 1–9 (5505 vs 3383, mean difference 1136, *p*_interaction_ < 0.001) (Table 5) (Fig. 2).

### PEDI Self-care

PEDI Self-care scores increased linearly after SDR (Fig. 1), with a significant improvement at 24 months, with an overall increase of 8.6 units (95% CI 5.0–12.3, *p* < 0.001). The improvement was seen most in GMFCS levels I, III, and IV (Table 4) (*p*_interaction_ < 0.001). The increase was greater in females (16.8 vs 12.4 units, *p*_interaction_ = 0.008). The increase was greater in age groups 10–18 (17.8 vs 12.7 units, *p*_interaction_ = 0.008), but not patients with dystonia (*p*_interaction_ = 0.61).

### PEDI Mobility

PEDI Mobility Scores increased linearly after SDR to 24 months after surgery (Table 3). The mean difference at 24 months was significant, with an overall increase of 4.4 units (95% CI 1.7–7.1), *p* = 0.002). There was a more significant increase in GMFCS levels I and IV (Table 4) (*p*_interaction_ = 0.021). Patients with dystonia had a greater increase (16.0 vs 2.3, *p*_interaction_ = 0.016). There was no difference in the increase for sex and age categories (*p*_interaction_ = 0.317 and 0.168 respectively).

### TUG

The TUG times decreased at 24 months from baseline (Table 3); however, the mean difference was not significant, with an overall decrease of 10.0 s (95% CI − 23.7 to 3.6, *p* = 0.147). There was no difference in the decrease between different GMFCS levels (Table 4) (*p*_interaction_ = 0.976). Patients aged 10–20 has a more significant time reduction (12.1 vs 9.6, *p*_interaction_ = 0.033), Females had a greater time reduction than males (14.5 vs 1.1, *p*_interaction_ = 0.043), as did patients with dystonia (25.2 vs 11.1, *p*_interaction_ = 0.007) (Table 5).

### Six-Min Walk test (6MWT)

Six-Min Walk test (6MWT) times decreased at 3 months after SDR, then increased to significantly higher levels than baseline at 24 months after surgery (Table 3), with an overall increase of 54.5 meters (95% CI 17.2–92.0), *p* = 0.005). There was no difference in the increase between different GMFCS levels (Table 4) (*p*_interaction_ = 0.984). There was no difference between the increase in age category (*p*_interaction_ = 0.116), sex (*p*_interaction_ = 0.551), or patients with dystonia (*p*_interaction_ =0.211) (Table 5).

### Gillette

Gillette FAQ scores increased linearly after SDR to 24 months after surgery (Table 3), and the mean difference at 24 months was significant, with an overall increase of 0.9 units (95% CI 0.3–1.4, *p* = 0.005). There was no difference in the increase between different GMFCS levels (Table 4) (*p*_interaction_ = 0.167). There was no difference in increase in sex, age category, or patients with dystonia (*p*_interaction_ = 0.934, 0.223, and 0.480 respectively) (Table 5).

### Modified Ashworth Scale (MAS)

MAS scores decreased after SDR to 24 months after surgery (Table 3). The mean difference was significant, with an overall decrease of 1.8 units (95% CI − 1.94 to − 1.61, *p* < 0.001). There was no difference in the decrease between different GMFCS levels (Table 4) (*p*_interaction_ = 0.748). There was no difference between the decrease in sex (*p*_interaction_ = 0.302), age category (*p*_interaction_ = 0.344), or patients with dystonia (*p*_interaction_ = 0.335) (Table 5).

Residuals were checked for normality to ensure model validity. In the models, there were no residuals that are univariate outliers. This does not bias the model or violate the assumptions.

## Discussion

This prospective single-center study showed that SDR increased GMFM-66 scores at 24 months after SDR that were statistically significant. This is in line with other smaller studies [24, 25], and a recent prospective, multi-center study that demonstrated a similar increase [8]. This further supports the meta-analysis of RCTs that demonstrated a greater improvement in GMFM-66 scores with SDR plus physiotherapy when compared to physiotherapy alone [12].

Our study adds that this increase may be exhibited across multiple gross, fine motor, and overall function tests (GMFM-66, PEDI Movement and Self-care, CPQoL, 6MWT, Gillette, and MAS scale). Cerebral palsy is the most common cause of physical disability in children worldwide, and improving overall function can increase social participation and improve quality of life [26]. Therefore, assessing interventions using a wide variety of performance measures is paramount.

There was no difference between GMFCS levels in the improvement in 5 out of 8 outcome measures. The use of SDR in GMFCS level IV patients has been explored by other studies [11]. with low patient numbers, and a recent systematic review reported lower complication rates in children with GMFCS grades IV and V who underwent SDR compared to ITB, and identified that SDR could potentially benefit these patients but cited a lack of evidence for this [27]. This indicates the need for larger sufficiently powered studies analyzing outcomes in this group.

There are no established minimally clinically important difference (MCID) reference ranges for most of the outcomes tested in children with cerebral palsy, but the improvement in GMFM-66 was above the “large” improvement value for each GMFCS class [28]. The improvement in 6MWT was significantly above the overall MCID reference in adults (> 50 m).

Our study also suggests that SDR may benefit patients aged 10–18 years old, as the improvement seen following SDR was either similar to younger children or greater in this age group. Older children have been previously thought to not benefit from SDR and even decline in function afterwards, with equivocal results observed in older children and adolescents undergoing the procedure [29]. These patients were not investigated by Summers et al. [8], and thus consideration of SDR as a useful treatment in this group could be investigated using appropriately selected cases. There was also no difference in the benefit experienced by patients with mixed spasticity and dystonia, which suggests that SDR with a view to improve spasticity specifically in these groups could potentially prove beneficial if enrolled and investigated prospectively.

The long-term effects of SDR are both dynamic and controversial [17, 18], and there is a need for nationwide prospective registries for those undergoing SDR in different countries to establish and evaluate long-term effects [30].

### Strengths of the study

Our prospective study included eight different scoring systems covering a wide array of activities assessing gross and fine motor function, quality of life, self-care, mobility, spasticity, and functional activity, providing a robust assessment of the effect of SDR on overall function and quality of life. Exploration to this extent has not been investigated in previous studies. Patients also had long-term follow-up lasting 24 months, with most patients having follow-up lasting 12 or 24 months. We also used a linear mixed effects model to account for patient attrition, varying attendance at follow-up clinics, and missing data, and ensure robust conclusions were met. The model allows the projected values of patients lost to follow-up to be accounted for in the mean values at each follow-up point. Data were also collected prospectively.

### Limitations of the study

Our study has several limitations. Firstly, our attrition rate is high, with only 30% of the original cohort having analysis available at 24 months. The reasons for this loss to follow-up are unclear, and could be due to patients not benefitting from SDR and subsequently not engaging in follow-up. It is not possible to quantify what magnitude this attrition has on our results, and this may impact long-term evaluations of the benefits of SDR, especially in atypical populations. The lack of sufficient follow-up in patients with GMFCS level I or V means we are unable to make any certain interpretation of the impact of SDR in these groups. Second, with lower powered data, it is more difficult to prove that a significant difference exists between subgroups compared to non-association. It therefore may be possible that the non-significant differences between subgroups (GMFCS level, age category, dystonia) may become visible with greater study power. This enforces the need for further studies with increased patient numbers in these populations. Third, we did not investigate other possible outcomes used that may be pertinent to cerebral palsy such as pain scores, or self-reported patient outcomes like patient goals. Finally, it is crucial to acknowledge that cerebral palsy is associated with other motor manifestations besides spasticity such as weakness and lack of motor control that are not likely to be relieved by SDR [26].

## Conclusions

Our study demonstrates that SDR may potentially lead to improvements in gross motor testing and overall function scores, mobility, quality of life scores, and self-care 24 months after SDR. Similar improvements in these parameters may be observed in older children, those with mixed spasticity and dystonia, and in each GMFCS level. This indicates that atypical patient populations may benefit from SDR if selected appropriately.

## Data Availability

Anonymized data are available (on reasonable request) from the corresponding author.
